# Rosuvastatin Enhances Angiogenesis via eNOS-Dependent Mobilization of Endothelial Progenitor Cells

**DOI:** 10.1371/journal.pone.0063126

**Published:** 2013-05-21

**Authors:** Junlan Zhou, Min Cheng, Yu-Hua Liao, Yu Hu, Min Wu, Qing Wang, Bo Qin, Hong Wang, Yan Zhu, Xiu-Mei Gao, David Goukassian, Ting C. Zhao, Yao-Liang Tang, Raj Kishore, Gangjian Qin

**Affiliations:** 1 Feinberg Cardiovascular Research Institute, Northwestern University Feinberg School of Medicine, Chicago, Illinois, United States of America; 2 Department of Cardiology, Northwestern University Feinberg School of Medicine, Chicago, Illinois, United States of America; 3 Department of Hematology, Union Hospital, Tongji Medical College, Huazhong University of Science and Technology, Wuhan, Hubei, P. R. China; 4 Department of Plastic Surgery, Tongji Hospital, Tongji Medical College, Huazhong University of Science and Technology, Wuhan, Hubei, P. R. China; 5 Key Laboratory of Molecular Biophysics of the Ministry of Education, College of Life Science and Technology, Center for Human Genome Research, Cardio-X Institute, Huazhong University of Science and Technology, Wuhan, Hubei, P. R. China; 6 Weinberg College of Arts and Sciences, Northwestern, Chicago, Illinois, United States of America; 7 Tianjin State Key Laboratory of Modern Chinese Medicine, Key Laboratory of Pharmacology of Traditional Chinese Medical Formulae, Ministry of Education, Tianjin University of Traditional Chinese Medicine, Tianjin, P. R. China; 8 CardioVascular Systems Biology, Steward St. Elizabeth's Medical Center, Tufts University School of Medicine, Boston, Massachusetts, United States of America; 9 Department of Surgery, Boston University Medical School, Roger William Medical Center, Providence, Rhode Island, United States of America; 10 Division of Cardiovascular Disease, Cardiovascular Research Center, University of Cincinnati, Cincinnati, Ohio, United States of America; University of Bristol, United Kingdom

## Abstract

Circulating endothelial progenitor cells (circEPCs) of bone marrow (BM) origin contribute to postnatal neovascularization and represent a potential therapeutic target for ischemic disease. Statins are beneficial for ischemia disease and have been implicated to increase neovascularization via mechanisms independent of lipid lowering. However, the effect of Statins on EPC function is not completely understood. Here we sought to investigate the effects of Rosuvastatin (Ros) on EPC mobilization and EPC-mediated neovascularization during ischemic injury. In a mouse model of surgically-induced hindlimb ischemia (HLI), treatment of mice with low dose (0.1 mg/kg) but not high dose (5 mg/kg) significantly increased capillary density and accelerated blood flow recovery, as compared to saline-treated group. When HLI was induced in mice that had received Tie2/LacZ BM transplantation, Ros treatment led a significantly larger amount of endothelial cells (ECs) of BM origin incorporated at ischemic sites than saline. After treatment of mice with a single low dose of Ros, circEPCs significantly increased from 2 h, peaked at 4 h, declined until 8 h. In a growth-factor reduced Matrigel plug-in assay, Ros treatment for 5 d induced endothelial lineage differentiation *in vivo*. Interestingly, the enhanced circEPCs and post-HLI neovascularization stimulated by Ros were blunted in mice deficient in endothelial nitric oxide synthase (eNOS), and Ros increased p-Akt/p-eNOS levels in EPCs *in vitro*, indicating these effects of Ros are dependent on eNOS activity. We conclude that Ros increases circEPCs and promotes their *de novo* differentiation through eNOS pathway.

## Introduction

Cardiovascular ischemic disease is the leading cause of morbidity and mortality worldwide and constitutes a major health burden. Recent clinical studies suggest that infusion of bone morrow (BM)-derived endothelial progenitor cells (EPCs) augments neovascularization of ischemic tissues and improve the therapeutic outcome [1]–[3]. In animal studies, EPCs have been shown to contribute to new vessel formation and tissue recovery by direct integration into injured vasculature [4]–[6], mediating favorable cell-to-cell contact [7], secreting paracrine factors [8], [9] and microparticles [10], and activating endogenous tissue stem/progenitor cells [11].

Statins are inhibitors of 3-hydroxyl-3-methyl coenzyme A reductase, the rate-limiting enzyme in cholesterol biosynthesis, and possess anti-inflammatory, anti-oxidant, anti-platelet and anti-fibrotic properties; thus, they are widely used in the treatment of dyslipidemia and the associated cardiovascular abnormalities [12]–[14]. Interestingly, considerable benefits have been demonstrated in statins’ clinical trials in patients with ischemic heart and peripheral disease, irrespective of the cholesterol concentration [15]–[18]. In fact, statins have been shown to stimulate angiogenesis by upregulation of the expression and activity of endothelial nitric oxide synthase (eNOS) [19]–[22]. eNOS is a key enzyme in the generation of nitric oxide in endothelial lineage cells, which not only contributes to angiogenesis induced by various stimuli [23] but also plays an important role in the mobilization of BM EPCs [24]–[26]; and studies from other laboratories suggest that statins enhance the functions of EPCs [27]–[29].

In this study, we have investigated the role of Rosuvastatin (Ros), a new and efficacious statin [30], [31], in the regulation of EPC function and ischemic angiogenesis by the use of BM transplantation (BMT) and surgical hindlimb ischemia (HLI) model in knockout and transgenic mice. Our results indicate that Ros increases EPC mobilization and promotes neovasculogenesis through an eNOS dependent mechanism.

## Materials and Methods

### Mice

Wild-type C57BL/6 and FVB/N mice and Tie2/LacZ transgenic mice (on FVB/N background) were purchased from The Jackson Laboratories (Bar Harbor, Maine). All animal work presented in this report was approved by the Institutional Animal Care and Use Committee (IACUC) of Northwestern University and performed in the barrier facilities of the Center for Comparative Medicine of the university.

### HLI Model

HLI was induced in 10- to 12-week-old male mice by surgical excision of the left femoral artery as described previously [5], [32]. The mice were anesthetized by inhaling Isoflurane™ delivered at 2–4% throughout the surgical procedure, and were injected subcutaneously with Metacam (1 mg/kg) as analgesic immediately after the surgery and then daily for the next 2 to 3 days. After the surgery, the mice were treated with Ros (AstraZeneca Pharmaceuticals, Cheshire, UK) and Simvastatin (Sigma-Aldrich) at serial doses for different time lengths. The blood flow recovery was monitored regularly with Laser Doppler perfusion imaging (LDPI) system (Moor Instruments, Wilmington, DE, USA) and expressed as perfusion ratio of ischemic/healthy (i.e. left/right) limbs. At 10 min before euthanasia, a 50 uL BS lectin (Vector Laboratories) was *i.v.* injected to facilitate identification of vasculature in the tissue sections. Then, the mice were euthanized by CO_2_ inhalation (primary method) and cervical dislocation (secondary method).

### Matrigel Plug-in Model

The plugs were created by subcutaneous (*s.c.*) injection of 300 µL mixture of growth factor-reduced Matrigel (BD Biosciences) with 2×10^6^ BM mononuclear cells (MNCs) at the back of mice with a G27 needle. After 5 days, the plugs were excised, examined microscopically, and stained with X-gal.

### Tie2/LacZ BM Transplantation (BMT)

Tie2/LacZ BMT was performed as described previously [4]. Briefly, donor Tie2/LacZ mouse BM MNCs were isolated with density centrifugation. The recipients, 8 week old male FVB/N mice, were irradiated (9 Gy), and each recipient received 2×10^6^ Tie2/LacZ BM MNCs via tail vein injection. One month later, the recipient mice were subject to surgical HLI and daily Ros injection. The engraftment routinely reached 80∼90% for FVB mice receiving Tie2/LacZ BM MNCs with the standardized protocol in our lab [5].

### Circulating EPC (circEPC) Culture Assay

CircEPCs were evaluated with a culture assay as previously described [5]. Briefly, peripheral blood (PB) MNCs were isolated from 300 µL blood with Histopaque-1083 (sigma) and seeded on 0.1% gelatin and 2.5 µg/ml rat vitronectin (sigma)-precoated 4 chamber slides containing 1 ml EBM-2 complete medium (EBM-2 CM). EBM-2 CM is EBM-2 basal medium plus the cytokine cocktail of EGM-2-MV SingleQuots (Clonetics, Inc**.,** San Diego, California). At day 4, a 2 µL of DiI-acLDL (Biomedical Technologies Inc., Massachusetts) was added to incubate for 4 h. The cells were then fixed in 1% paraformaldehyde (PFA) and counter-stained with 1% (v/v) Isolectin B4-FITC (Vector Laboratories, Inc., California). The adherent cells positive for both Isolectin B4-FITC binding and DiI-acLDL uptake were considered EPCs, which reflect the initial circulating EPCs present in the PB MNCs. Randomly chosen 12∼20 fields with 200× magnification were counted by blinded investigators under a fluorescent microscope.

### X-gal Staining and Immunohistochemistry

Tissues or Matrigel plugs were fixed either in 4% PFA or 100% methanol. Enzymatic staining for X-gal and immunohistochemical staining with antibodies for BS Lectin (Vector laboratories, Burlingame, CA) and β-Gal (Cell Signaling Technology, Beverly, MA) were performed as previously described [5], [32], [33].

### Western Blotting

EPCs were isolated and cultured from BM MNCs for 7 days with a standardized protocol established in our lab [5], and then treated with different concentrations of Ros for 30 min. Western blotting analyses with antibodies for eNOS, phospho-eNOS (Ser1177), Akt, and phospho-Akt (Ser473) (Cell Signaling Technology) were performed as previously described [34]–[36]. Band intensities were determined densitometrically with Image J software.

### Statistical Analysis

Data are presented as average ± SEM. Unpaired Student’s *t* test was used for the significance of differences. P<0.05 was considered significant.

## Results

### Ros Enhances Neovascularization

**Figure 1:**
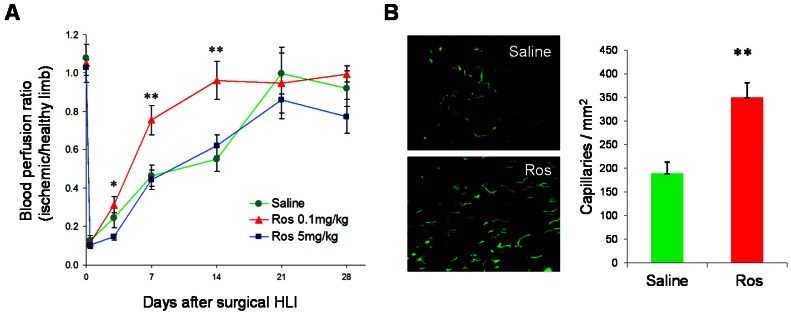
Ros enhances ischemic neovascularization. (**A**) C57BL/6 male mice were rendered surgical hindlimb ischemia. Ros at low dose (0.1 mg/kg), high dose (5 mg/kg), or saline was injected daily, and blood flow recovery was monitored by LDPI at days 3, 5, 7, 14, 21, and 28 after the surgery. n = 8, *P<0.05, **P<0.01 vs. Saline. (**B**) Representative micrographs of BS lectin staining (*left panel*, 200× original magnification) and quantification of capillary densities (*right panel*) in the limb tissues at day 14. n = 8, **P<0.01 vs. Saline.

To investigate the effect of Ros on neovascularization after ischemic injury, we induced acute ischemia in C57BL/6 mice by surgically removing the left femoral artery. Ros at low dose (0.1 mg/kg), high dose (5 mg/kg), or Saline control was injected subcutaneously (*s.c.*) daily from day 0 to day 28 following the surgery. The low-dose but not high-dose group of mice demonstrated more rapid blood flow recovery than the control group at days 3, 7 and 14 (Figure 1A). Low dose Ros treatment also increased capillary density in the ischemic limb (Figure 1B). Thus, we continued our investigations of Ros at low dose for the following *in vivo* studies.

### Ros Increases Incorporation of BM-derived EPCs at Sites of Ischemic Injury

**Figure 2:**
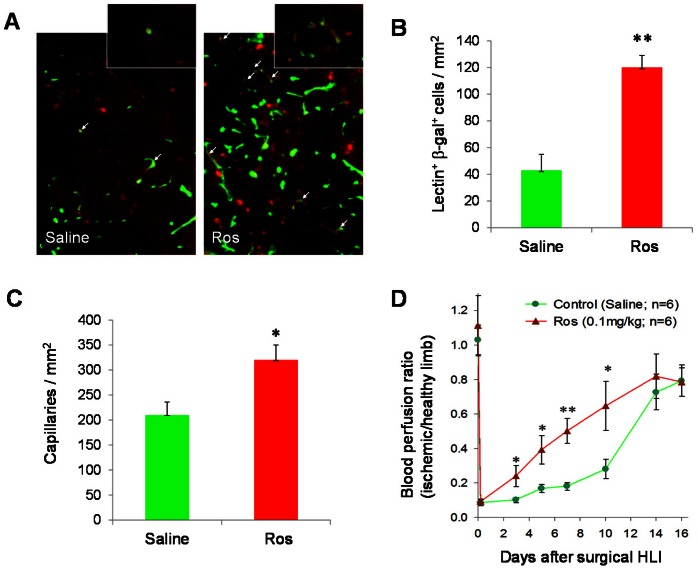
Ros increases BM-derived EPCs in neovascularization. BM MNCs isolated from Tie2/LacZ mice were used to transplant lethally irradiated syngeneic FVB/NJ mice. One month later, HLI was induced in the recipient mice, and Ros (0.1 mg/kg) was *s.c.* injected daily. (**A–C**) At day 14 after HLI, mice were injected with BS lectin and 10 min later, euthanized. The ischemic tissues were stained immunofluorescently with BS lectin (FITC, green) and β-gal (PE, red) to indicate vasculature and BM-derived cells, respectively. (**A**) Representative images of immunofluorescent double staining. White arrows indicate BS lectin and β-gal double positive cells. 400× original magnification. (**B**) Quantification of BM-derived EPCs incorporated in the neovasculature (i.e., double positive cells) in the ischemic limb. (**C**) Quantification of capillary densities (BS lectin-FITC positive cells). n = 6, *P<0.05, **P<0.01 vs. Saline. (**D**) Blood flow was monitored with LDPI at days 3, 5, 7, 10, 14, and 16 following HLI. *P<0.05, **P<0.01 vs. Saline.

To assess the effect of Ros on BM EPC contribution to neovascularization, we performed BM transplantation (BMT) to reconstitute the BM of lethally-irradiated WT FVB/N mice with BM MNCs from background-matched Tie2/LacZ mice. One month later, recipient mice with >90% engraftment were chosen to receive surgical HLI and treatment with Ros or saline. Ros treatment led to a significantly greater number of BM-derived ECs (as shown by BS lectin and β-gal double positive cells) incorporated at ischemic sites (Figures 2A and 2B), which were associated with an increased capillary density and accelerated blood flow recovery in the ischemic limb (Figures 2C and 2D), although recovery of the mice on FVB/N background were generally slower than those on C57BL/6 background (Figure 1A).

### Ros Increases the Levels of circEPCs and Endothelial Lineage Differentiation from BM MNCs

To understand the cellular mechanism by which Ros increases EPC incorporation into neovascularization, we analyzed the effect of Ros on the level of circEPCs (i.e., EPC mobilization). Different doses of Ros or saline were *s.c.* injected into WT C57BL/6 mice and the amount of circEPC were evaluated 24 h later with a EPC culture assay [5]. CircEPC number peaked at a dose of 0.1 mg/kg, being 6 times higher than untreated mice and 3 times higher than phVEGF treated mice (Figure 2A). Moreover, after single injection of 0.1 mg/kg Ros or 0.2 mg/kg Simvastatin (Sim) [37], circEPCs significantly increased from 2 h, peaked at 4 h, declined until 8 h (Figure 2B). These results suggest that Statins mobilize EPCs quickly and in a dose dependent manner, and that the enhanced neovascularization in Ros-treated mice may be attributable, at least partly, to an increase in EPC mobilization.

**Figure 3:**
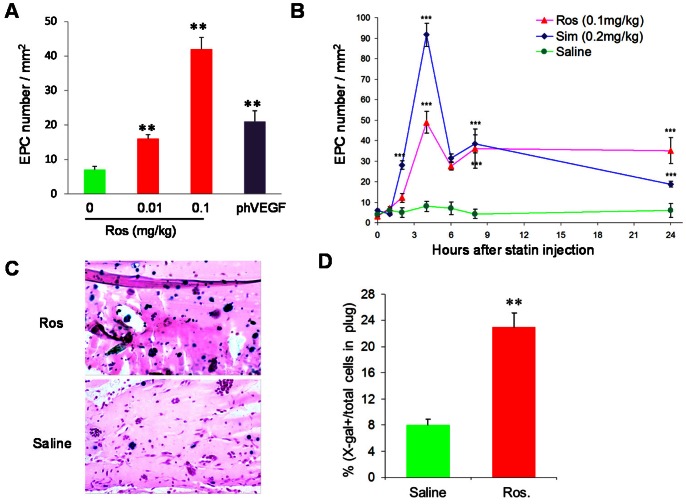
Ros increases circEPCs and promotes endothelial lineage differentiation. (**A**) C57BL/6 male mice received daily *s.c.* injections of different doses of Ros for 7 days. Twenty-four hours after last injection, PB MNCs were collected, and circEPCs were evaluated with a culture assay. One group received an intramuscular injection of 200 ug pVEGF165 plasmid as positive control. n = 4, **P<0.001 vs. Saline. (**B**) The kinetics of PB circEPCs within 24 h after *s.c.* injection of a single dose of Ros (0.1 mg/kg) or Sim (0.2 mg/kg). n = 4, ***P<0.001 vs. Saline. (**C–D**) Mice were *s.c.* injected with a mixture of 300 uL growth factor-reduced Matrigel and 2×10^6^ Tie2/LacZ BM MNCs and then s.c. injected with Ros (0.1 mg/kg) or saline daily for 5 days. The Matrigel plugs were then removed, fixed, and stained in X-gal solution. (**C**) Representative images. (**D**) β-gal positive cells were quantified and expressed as percentage of total cells, n = 4, **P<0.01 vs. Saline.

Although the increase in new vessel formation could be the result of this mechanism alone, we also considered the possibility that Ros could enhance differentiation of EPCs. To test this hypothesis we performed experiments in which ∼5×10^6^ undifferentiated BM MNCs isolated from Tie2/LacZ mice were mixed in growth factor-reduced Matrigel and *s.c.* implanted into background-matched WT mice that receive daily injection of either 0.1 mg/kg Ros or saline for 5 days. Since growth factor-reduced Matrigel does not provide a sufficient substrate to support the growth of new vessels, this model provided us with the opportunity to quantify EPC differentiation *in vivo*, defined by the advent of Tie2 driven LacZ expression in the population of unselected BM MNCs. As shown in Figures 3C and 3D, a significantly higher ratio of X-gal (+) to total cells was found in the Matrigel plugs in Ros-treated mice than in saline-treated mice. Since an equal number of BM MNCs were implanted in both groups, these data indicate that Ros enhances *de novo* EPC differentiation *in vivo*.

### The Ros-induced Enhancement of EPC Mobilization and Blood Flow Recovery is dependent on eNOS pathway

**Figure 4:**
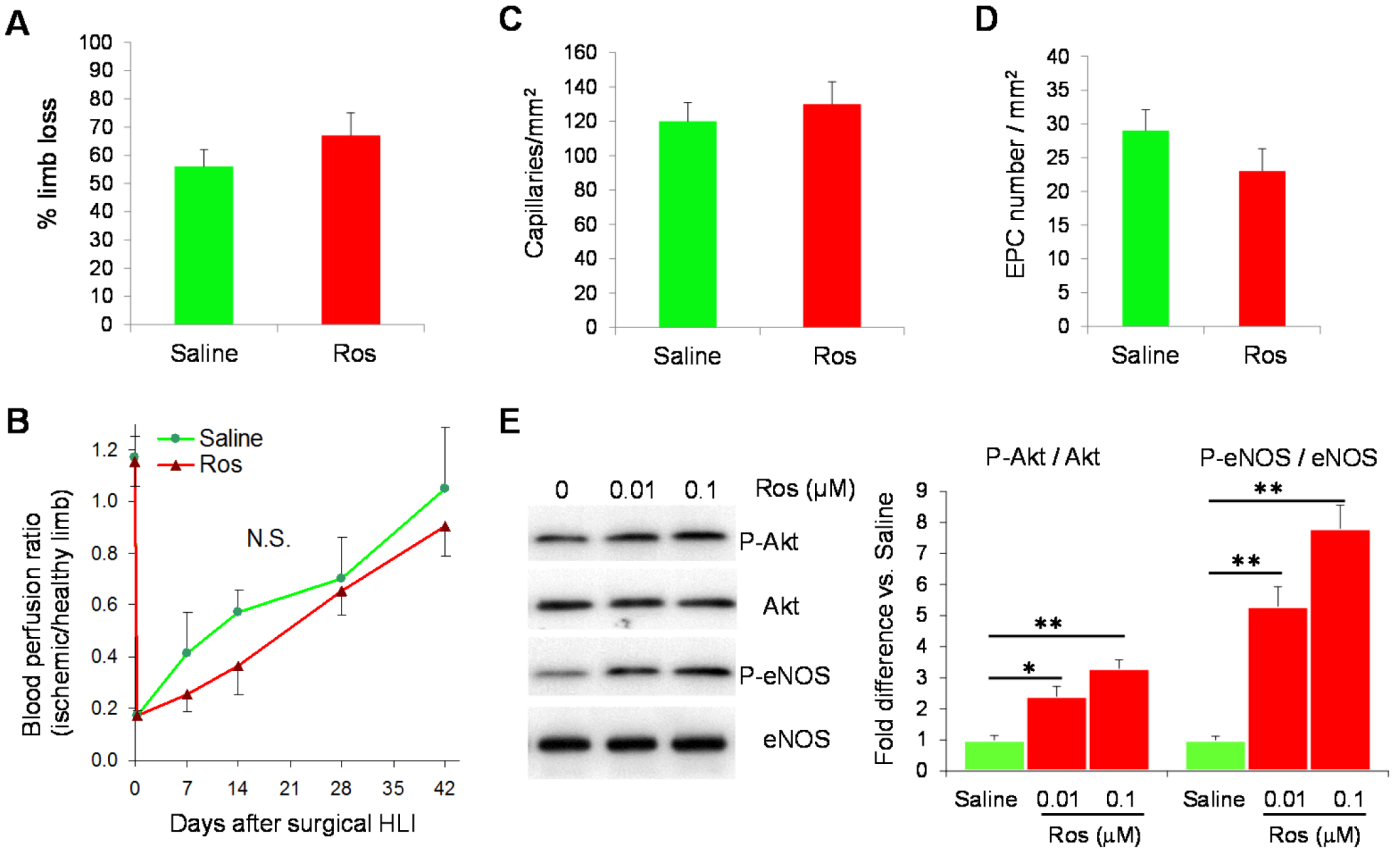
The Ros-mediated enhancement of EPC mobilization and neovascularization is dependent on eNOS expression. (**A–C**) eNOS^−/−^ mice were rendered surgical HLI and *s.c.* injected daily with Ros (0.1 mg/kg) or saline. (**A**) The rate of limb loss, n = 18. (**B**) Blood flow assessments with LDPI in mice without limb loss at day 7, 14, 28, and 42 after the surgery. (**C**) In a separate experiment, capillary density in the ischemic limb of day 14 was evaluated with BS lectin, n = 6. (**D**) eNOS^−/−^ mice received daily s.c. injections of Ros (0.1 mg/kg) for 7 d. PB MNCs were collected 24 h after the last injection, and circEPCs were assayed with the culture assay, n = 4. (**E**) EPCs were cultured from BM MNCs of WT mice for 7 days, and then treated with different doses of Ros for 30 min. The cells were lysed and the levels of phospho-Akt, Akt, phospho-eNOS, and eNOS were analyzed by Western blotting. *Left panel,* representative Western blotting. *Right panel*, the levels of phospho-Akt and phospho-eNOS were normalized to Akt and eNOS, respectively, and expressed relative to the values in saline-treated group. n = 4, *P<0.05, **P<0.01 vs. Saline.

Because eNOS mediates several beneficial effects of statins for vascular protection [38] and has also been shown to be essential for BM EPC mobilization [39], we investigated whether eNOS pathway is a potential molecular mechanism by which Ros mobilizes BM EPCs and enhances neovascularization. In eNOS-null (eNOS^−/−^) mice, HLI surgery led to a significant amount of limb loss; however, there is no significantly difference between Ros and saline treatments (Figure 4A). The blood perfusions in the preserved hindlimbs were also similar between the two treatment groups (Figure 4B). Moreover, the increase in capillary density in the ischemic limbs and the elevated levels of circEPCs induced by Ros were also blunted in the eNOS^−/−^ mice (Figures 4C and 4D), indicating that these effects of Ros are dependent on a normal eNOS activity. Consistently, Ros treatment of cultured EPCs increased levels of phosphorylated Akt and eNOS (Figure 4E).

## Discussion

In this study, we have demonstrated that Ros potently mobilizes EPCs, promotes EPC *de novo* differentiation, and significantly enhances neovascularization and blood flow recovery after ischemic limb injury. In addition, our results indicate that the beneficial effects of Ros on EPC mobilization and neovascularization are dependent on eNOS activity.

Similar to other statins [40]–[42], Ros demonstrates a biphasic effect on neovasculogenesis; however, the precise mechanism is currently not clear. Low doses of statins have been shown to enhance neovasculogenesis by activating endothelial Ras, promoting Akt and eNOS phosphorylation [40], [43], and upregulating eNOS expression [44], [45], whereas high statin doses may decrease protein prenylation in ECs, inhibit cell growth, and induce apoptosis [40].These biphasic activities of statins on EC biology can potentially be explained by the properties of the biosynthetic pathways that originate from mevalonic acid [46], because in addition to cholesterol, mevalonic acid is an essential precursor for several cellular components including ubiquinone, isopentenylated transfer RNAs, and prenylated proteins. Mevalonate-derived intermediates have a higher affinity for the enzymes that catalyse non-sterol product formation than for the cholesterol biosynthetic enzymes. Therefore, low doses of statins may predominantly affect cholesterol synthesis and not interfere with the biosynthesis of non-sterol products that are required for cellular housekeeping functions, and only at higher statin doses may a significant inhibition of non-sterol product synthesis occur.

Ros is by far the most efficacious statin [31], [47]; nevertheless, the JUPITER study, despite reducing CVD and overall mortality, highlighted an increase in new onset diabetes in the Ros treated arm [47]. More recently, the increase in the incidence of diabetes during statins trials has been confirmed by many meta-analyses of the randomized controlled trials and appears to be associated with a higher statin dosage [48], [49]. Our study suggests that a lower dose may be more favorable, at least in patients with diabetes or diabetes associated risk factors.

It is exciting that low dose of Ros significantly enhances EPC mobilization and recruitment to the site of neovascularization. Stem cell mobilization involves complicated adhesive interactions or cross-talks between stem cells and BM microenvironment [34], [35], [50], [51]. For example, G-CSF acts not directly on hematopoietic stem cells (HSCs) but via receptors on cells of the BM stroma [52], while cleavage of VCAM-1 with neutrophil protease was also involved in HSC mobilization [53]. The kinetics of HSC mobilization with chemokines versus cytokines range from a few minutes (with chemokines) to several days (with hematopoietic growth factors) [51]. The kinetics of EPC mobilization, however, varies considerably. Vascular trauma and ischemia was reported to induce rapid but transient EPC mobilization [54]. Previous study from our institute demonstrated that circEPCs reached a peak at day 7 after surgical ischemia in rabbits [55]. The mobilization of EPCs in nude mice with Ad-VEGF injection is rapid, peaks at 2–3 days while Angiopoietin-1 exerts a delay EPC mobilization as compared to VEGF, peaks at 2 weeks [56]. Other researchers reported that peripheral circEPC numbers increased gradually, reached peak with 4 weeks treatment of statins in mouse models and in patients with coronary diseases [29], [57]. Our data further show that the kinetics of Ros-induced EPC mobilization is more resembling to that of “chemokine-type”. Further investigations are underway to determine how high Ros doses affect EPC mobilization and neovasculogenesis.

We found that the enhanced circEPCs and post-HLI angiogenesis stimulated by Ros were blunted in eNOS^−/−^ mice, suggesting an essential role of eNOS. Because Ros upregulates eNOS in both EPCs and ECs, it is difficult to dissect the extent to which the angiogenic effect of the statin is dependent on EPCs. Our attempt to reconstitute WT mouse BM with that of eNOS^−/−^ mice was with low efficiency presumably due to the indispensable role of eNOS for a successful BMT. Currently, it is not completely clear how eNOS is activated (i.e., phosphorylated) by Ros. Since PI3K/Akt pathway has been shown essential to EPC mobilization, migration, proliferation, and survival [37], [57] and our results indicate that Ros also mediates Akt phosphorylation, it is therefore likely that eNOS be a down-stream mediator of Akt [58]–[60]; this however, remain to be investigated in our future study.

In summary, our study demonstrates that Ros at a lower dose promotes ischemic neovascularization via eNOS-dependent EPC mobilization. Thus, optimization of Ros dose may maximize the effect of Ros for the prevention and treatment of ischemic disease.
